# A Systematic Review Examining Associations between Cardiovascular Conditions and Driving Outcomes among Older Drivers

**DOI:** 10.3390/geriatrics5020027

**Published:** 2020-04-28

**Authors:** Ganesh M. Babulal, Ramana Kolady, Sarah H. Stout, Catherine M. Roe

**Affiliations:** 1 Department of Neurology, Washington University School of Medicine, St. Louis, MO 63110, USA; 2 Cupertino High School, Cupertino, CA 95014, USA

**Keywords:** driving, vascular condition, stroke, white matter hyperintensities, heart failure, dementia, naturalistic methods, cessation

## Abstract

There is a vast literature on stroke as a cardiovascular disease and driving outcomes, however little is known about other cardiovascular conditions and driving. The purpose of this review is to examine the literature for studies assessing the effect of non-stroke, vascular conditions on daily driving, reported crash risk and driving decline in older adult drivers as captured by naturalistic methodologies. A systematic review of Embase, Ovid and Scopus Plus examined articles on driving and vascular conditions among older adults. A search yielded 443 articles and, following two screenings, no articles remained that focused on non-stroke, vascular conditions and naturalistic driving. As a result, this review examined non-stroke, vascular conditions in nine driving studies of older adults that used road testing, driving simulators and self-report measures. These studies fell into three categories—heart failure, vascular dementia and white matter hyperintensities/leukoaraiosis. The combined findings of the studies suggest that heart failure, vascular dementia and white matter hyperintensities (WMH) negatively impact driving performance and contribute to driving cessation among older adults. Future research should examine cardiovascular risk factors like hypertension, hypercholesterolemia, myocardial infraction or atherosclerosis using naturalistic driving measurement, as well as traditional measures, in order to more fully characterize how these conditions impact older adult driving.

## 1. Introduction

In 2016, among the pool of 42 million licensed drivers age ≥ 65, there were 290,000 injures and over 7400 deaths resulting from motor vehicle crashes in the Unites States (US) [1]. Compared to younger drivers (ages 25–64), older drivers have a slower reaction time and are more likely to cause a crash [2]. Since older adults are driving longer, crashes and deaths (driver, pedestrians) will continue to increase based on the volume of active drivers. An increased crash risk among aging drivers has been attributed to a heterogeneity of factors, including visual impairments, medications that impair the central nervous system, cardiovascular conditions, cognitive impairment and Alzheimer’s disease (AD) [3,4,5,6]. Conversely, older adults tend to restrict their driving miles and space [7,8,9] suggesting that they may recognize to some extent that they are at greater risk of crashes as they age. Unfortunately, however, self-regulation is a poor compensatory approach to reduce crash risk; and instead, avoidance of driving is a proven risk factor for crashes (past/future) and impaired driving [10,11,12]. 

Cerebrovascular disease (CVD) encompasses a number of conditions which affect the blood flow to and structures (blood vessels) of, the brain. Stroke is the most common CVD with different types including ischemic, transient ischemic attack and hemorrhagic. With over 795,000 annual cases of new or recurrent stroke in the US and $34 billion in direct and indirect healthcare costs, stroke is a leading cause of long term disability and limitation in mobility [13]. A number of modifiable risk factors can increase CVD prevalence, including hypertension, diabetes, hypercholesterolemia, obesity and smoking. Most of the CVD burden stemming from these risk factors is acquired among adults between the ages of 35 to 64 years [14]. As a consequence of brain injuries caused by stroke, a person may experience decline in cognitive functioning, eventually develop dementia and may have impairments in instrumental activities of daily living (e.g., driving) [15,16,17]. 

Compared to CVD, cerebral small vessel disease (CSVD) is a colloquial umbrella of conditions impacting arteries, arterioles, venules and capillaries [18]. Imaging modalities, specifically using magnetic resonance imaging (MRI) is able to identify microbleeds, atrophy, infracts, lacunas and leukoaraiosis or white matter hyperintensities (WMH) [19]. For example, leukoaraiosis/WMH are pathological changes in the white matter causes by hypoxia-ischemia due to changes/damage in the walls of small vessels [20]. Leukoaraiosis and other CSVD are common in patients with stroke [21,22,23] and are also associated with cognitive impairment, depression, gait disturbances and dementia [24,25,26,27]. While CSVD influence other conditions, it is unclear if there is any direct impact on driving.

There is a significant body of work including, systematic reviews [28,29,30,31,32] on stroke and driving secondary to the sensory and motor impairments that may persist post-acute, subacute and rehabilitation services. These findings are generally derived from a single measurement in time—results may quantify the risk of failing a road test, the risk for crashing or making specific operational errors and the likelihood of driving cessation. Most of this literature in stroke has employed road tests, driving simulators and crash/accident history for the driving outcomes. These outcomes are methodological mainstays and provide insight into driving performance via controlled conditions and event-based data points. Yet they are impacted by objectivity, replication, generalizability from single site-specific measure, Hawthorne effect and confound of anxiety [7,33,34]. These limitations can be supplemented by employing naturalistic driving methodologies (e.g., in-vehicle dataloggers), which better capture longitudinal day-to-day driving behavior of older adults in their own environments. Naturalistic data may better provide a gestalt of driving decline due to a particular condition or improvements in daily driving due to a specific intervention.

It is unclear whether research in vascular conditions (e.g., heart attack, vascular dementia, CSVD) and driving outcomes employ naturalistic driving outcomes to better understand driving behavior. The purpose of this review is to examine the extant literature for studies assessing the effect of non-stroke, vascular conditions’ on daily driving as captured by naturalistic methodologies and determine whether there is an increased risk for crashes or driving decline among older adult drivers with a non-stroke, vascular condition.

## 2. Methods

### 2.1. Literature Search Strategy

We employed the PICO (Population, Intervention, Comparison, Outcome,) framework to determine the overall structure of this systematic review. Our population/participants of interest included older adult drivers, which was defined here as those of 60 years of age or older. Use of interventions/treatment was not a focus of this review; however, if an intervention was used, it was examined in the context of driving. We were also interested in studies that compared persons with symptomatic, vascular conditions to controls. The outcome of interest (driving) could vary but was required to be contextually relevant to driving as an activity and could encapsulate driving performance, crashes, cessation, natural driving, fitness to drive and use of an automobile. 

The published literature was searched using strategies designed in consultation with a clinical librarian in our medical school for the concepts of vascular conditions, including stroke and vascular dementia, driving and elderly or older aged adults. The search strategies were created using a combination of database specific and controlled vocabulary terms and keywords and were executed in Embase 1947-, Ovid-Medline 1946- and Scopus 1823-. Each database only examined the title, abstract and indexing fields of a specific citation record. Results were limited to English language using database-supplied filters. 

All searches were completed in March 2020. A complete search strategy is provided for Embase.com as an example—(‘cerebrovascular accident’/exp OR ‘white matter hyperintensities’/exp OR ‘white matter’/de OR ‘hypertension’/exp OR ‘hypercholesterolemia’/exp OR ‘heart infarction’/exp OR ‘congestive heart failure’/exp OR ‘multiinfarct dementia’/exp OR stroke *:ti,ab OR ‘cerebrovascular accident’:ti,ab OR ‘cerebrovascular apoplexy’:ti,ab OR ‘cerebrovascular lesion’:ti,ab OR ‘brain accident’:ti,ab OR ‘brain attack’:ti,ab OR ‘brain insultus’:ti,ab OR ‘brain vascular accident’:ti,ab OR ‘cerebrovascular failure’:ti,ab OR ‘cerebrovascular injury’:ti,ab OR ‘white matter hyperintensities’:ti,ab OR leukoaraiosis:ti,ab OR ‘white matter’:ti,ab OR hypertension:ti,ab OR hypertensive:ti,ab OR ‘high blood pressure’:ti,ab OR hypercholesterolemia:ti,ab OR ‘high cholesterol’:ti,ab OR ‘elevated cholesterol’:ti,ab OR hypercholesterinaemia:ti,ab OR hypercholesterinemia:ti,ab OR hypercholesterolaemia:ti,ab OR ‘heart attack’:ti,ab OR ‘myocardial infarct *’:ti,ab OR ‘heart infarct *’:ti,ab OR ‘cardiac infarct *’:ti,ab OR ‘myocardium infarct *’:ti,ab OR ‘subendocardial infarct *’:ti,ab OR ‘cardiovascular stroke’:ti,ab OR ‘congestive heart failure’:ti,ab OR ‘congestive cardiac failure’:ti,ab OR ‘congestive heart insufficiency’:ti,ab OR ‘CHF’:ti,ab OR ‘vascular dementia’:ti,ab OR ‘arteriosclerotic dementia’:ti,ab OR ‘Binswanger disease’:ti,ab OR ‘Binswanger encephalopathy’:ti,ab OR ‘subcortical leukoencephalopathy *’:ti,ab OR ‘multi-infarct dementia’:ti,ab OR ‘multiinfarct dementia’:ti,ab) AND (‘car driving’/exp OR ‘driving cessation’ OR ‘natural * driving’ OR ‘natural * drive’ OR ((drive OR driving OR use) NEAR/6 (car OR auto OR automobile * OR truck * OR van OR vans OR vehicle *))) AND (‘aged’/exp OR elderly:ti,ab OR aged:ti,ab OR older:ti,ab OR “senior citizen”:ti,ab OR geriatric:ti,ab OR senescent:ti,ab) AND [english]/lim.

### 2.2. Inclusion/Exclusion Criteria 

Study titles and abstracts were reviewed and screened for specific criteria. A study was excluded if it (1) did not have a focus on older adults (age ≥ 60 years), (2) there was no reference to a vascular condition or driving in the title or abstract, (3) did not encompass driving as the outcome or activity, (4) was a review, qualitative in nature, a case study, a text book, a white paper or a position paper, (5) used a sample of professional drivers (e.g., taxi, bus, trucks/lorries) and (6) focused on validation of a scale, tool or instrument. 

### 2.3. Data Extraction and Synthesis

Duplicate citations were removed across the three databases before extraction. Results were exported to EndNote X9 reference manager with a combined library of all results and a separate library based on results returned by individual databases. Each study was examined based on the inclusion/exclusion criteria in Section 2.2. Next, a secondary screen was conducted among the abstracts of the remaining studies and the studies were arranged into four categories—(1) both driving and a single vascular condition were not clearly identified in the abstract, (2) a study on general aging and any driving outcome with some mention of vascular conditions, typically as a covariate, (3) a study on stroke as a vascular condition and naturalistic driving outcome and (4) a study on a vascular condition other than stroke and naturalistic driving. Following this secondary screen, the full-texts were retrieved for the remaining publications to be included in this analysis. 

## 3. Results

**Figure 1:**
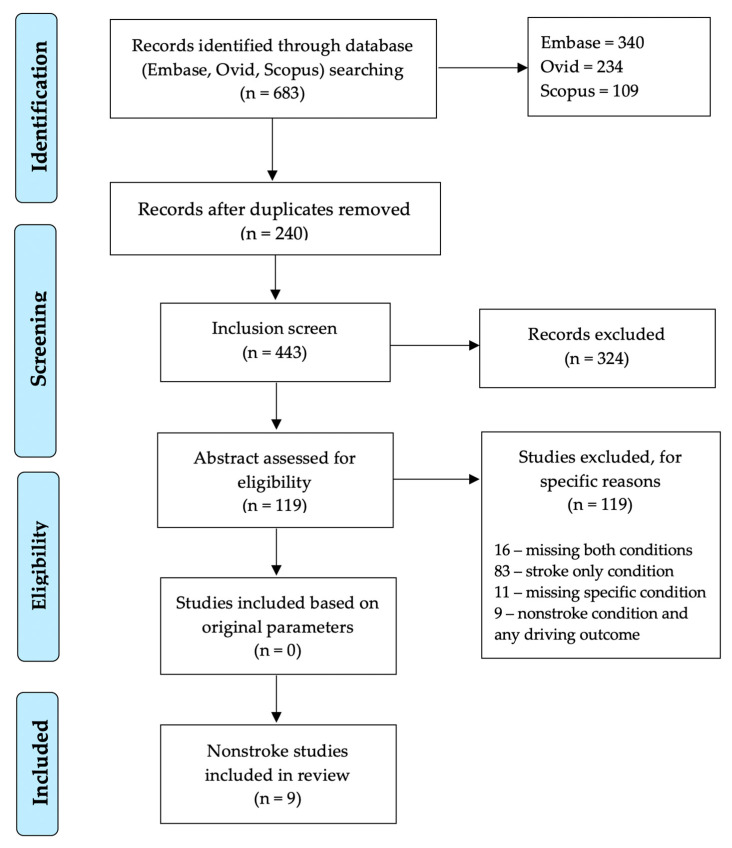
PRISMA flow diagram of studies included in review.

Across the three databases, 683 results were initially retrieved and after removal of duplicates (240), the final search yielded 443 articles (Figure 1). After the first screen of inclusion/exclusion criteria was applied, 119 unique citations remained. Of the excluded citations (324), 28 were qualitative in nature with the majority describing a case study, 33 focused on professional drivers, 10 had samples that were less than age 60, 11 were about development of scales, one was an animal/mice study and the remaining studies discussed either a vascular condition or driving but not both. After the second screen was applied, no articles remained. Of the citations excluded by the second screen (119), 16 did not include both a driving outcome and a vascular condition, 11 had a general focus on older adults and driving without a specific vascular condition, 83 involved stroke and a driving outcome (road test, simulator) and the remaining 9 examined a vascular condition other than stroke and some driving outcome. 

None of the studies on stroke (83) or non-stroke vascular conditions (9) examined driving as an outcome using a naturalistic methodology (e.g., dataloggers, camera). A majority of the articles on stroke examined driving via performance on a road test, simulator or crash history, while the remaining assessed driving cessation via questionnaires. Given the expansive literature on stroke and driving, we elected to examine the nine non-stroke studies and their driving outcomes. Of the nine non-stroke studies, five examined heart failure, two focused on vascular dementia, two assessed WMH/leukoaraiosis (Table 1). Four studies evaluated driving performance on a driving simulator, three via driving cessation on a questionnaire per self-report and two studies used a road test. 

### 3.1. Heart Failure 

Across three driving simulator studies, Alosco and colleagues (2013, 2015a, 2015b) found that older adults with heart failure (n = 18) had (1) poorer performance on attention/executive function tests and simulated driving compared to younger adults [35], (2) more WMH and reduced whole brain volume were associated with poor driving performance [36] and (3) decreased physical fitness was associated with poor driving performance [37]. During the simulation scenarios, collisions and missed stop signs were consistent markers of impaired driving performance. The latter two studies investigated the impact of heart failure on driving cessation. In a large community sample of older drivers (5383), Sim et al. (2011)) found that patients with heart failure were more likely to stop driving over nine years compared to those without health failure after adjusting for multiple covariates [38]. Among those with heart failure (n = 839), older age (<74), female sex, reduced physical fitness, visual problems and stroke were independent predictors of driving cessation. A similar study (n = 850) by Fausto and colleagues (2017) found that older adults with a diagnosis of heart failure (n = 29) were more three times more likely to stop driving over the course of three years compared to those without health failure. However, this main effect was not statistically significant when performance on the Useful Field of View, a visuospatial task was added in a second model and age along with several neuropsychological tests were added to a third model.

### 3.2. Vascular Dementia 

Only two studies had explicit focus on vascular dementia. Fitten et al. (1995) examined performance on a road test across several groups including AD (n = 13), vascular dementia (n = 12), diabetes (n = 15), older controls (n = 24) and younger controls (n = 16) [39]. Older adults with a diagnosis of AD or vascular dementia had poorer performance on the road test. Compared to those with AD, patients with vascular dementia has less severe impairment but had more variability within the group on the road test score. A more recent study by Piersma and colleagues (2018) examined differences on a road test and driving simulator across vascular dementia (n = 14), frontotemporal dementia (n = 12) and dementia with Lewy bodies (n = 8) groups [40]. Approximately 29% with vascular dementia failed the road test compared to 42% with frontotemporal dementia and 62% with dementia with Lewy bodies. Compared to the other two groups, older adults with vascular dementia made more errors (number of collisions) on the driving simulation and had poorer performance on neuropsychological tests. 

### 3.3. WMH/Leukoaraiosis

The final two studies using MRI examined WMH/leukoaraiosis on driving. Nakano and colleagues (2014) assessed performance on a road test across a young control group (n = 9), older group without subcortical WMH (n = 11) and an older group with multiple subcortical WMH (n = 12) [41]. The older groups made more overall errors during normal operation and were more distracted by external sound disturbance compared to the younger group. However, the older group with WMH made more critical operation errors and steered less smoothly compared to the older group without WMH. Jang et al. (2018), examined the association between mild (n = 389), moderate (n = 116), severe (n = 35) WMH rating and driving cessation among older drivers [42]. WMH was directly associated with driving cessation cross-sectionally, while adjusting for age, sex, education, cognitive and motor functioning. Most importantly, increasing severity of WMH was associated with faster change from currently driving to driving cessation over time.

## 4. Discussion

This systematic review set out to assess the literature to determine whether vascular conditions other than stroke impacted daily driving as assessed by naturalistic methodologies among older adult drivers. No study met the inclusion criteria and screening for this systematic review when compared to the study’s purpose outlined in the introduction. The common factor influencing these results was that no study used driving outcome data as captured by technology from a personal vehicle (e.g., data loggers, cameras). Among the studies, driving outcomes ranged across road tests, driving simulators and self-report; and as expected, stroke was the most common CVD. The nine studies ultimately reviewed used road tests and driving simulators for performance and self-report information regarding driving cessation.

Of the nine full texts retrieved for this systematic review, more than half of the studies examined driving performance and cessation in older adults with heart failure. This large proportion of studies is likely due the fact that heart failure affects approximately 6.5 million adults contributing to 1 in 8 deaths in the United States and increases in prevalence with age [13]. The consensus across these studies suggests that patients with heart failure have poorer performance on simulated driving and are more likely to stop driving earlier compared to those without heart failure. The two studies on vascular dementia were 23 years apart but summatively posited that older adults with vascular dementia are more likely to fail a road test and make more errors on a driving simulation. The final two studies used structural MRI indices to rate WMH and found that older adults with WMH exhibited worse driving performance on a road test and faster driving cessation. Taken together, these findings suggest that heart failure, vascular dementia and WMH negatively impact driving performance and contribute to driving cessation among older adults. We did not identify any studies that examined the effect of hypertension, hypercholesterolemia, myocardial infraction or atherosclerosis on any driving outcome despite their inclusion in the search strategy. 

There were some limitations among these studies. If a study did not use a sample from an established cohort (e.g., Cardiovascular Heart Study), the participant pool was small, typically below 30 individuals. Additionally, only three studies examined data longitudinally in their analyses. The combination of small sample sizes and cross-sectional analyses limits the generalizability of the findings. One of the two studies on WMH/leukoaraiosis had minimal information on methodology regarding MRI acquisition, reading and lesion identification of scans (e.g., periventricular vs deep) and translation into an overall rating [41]. Additionally, determination of the cognitive status of participants varied across studies, with most relying on the Mini Mental Status Examination [43] for a screening measure, without any full cognitive evaluation. Since older adults are at higher risk for cognitive impairment and dementia, it is essential to classify the cognitive status of the sample. Only two studies used more formative, diagnostic measures like the Clinical Dementia Rating [44] to determine cognitive status. Neuropsychological tests were used to evaluate cognitive functioning with considerable variability on how results were utilized in analyses. 

Given that the population of older adults is expected to double to 88 million by 2050 in the United States, a quarter of all drivers will be 65 and older at that time [1,45]. Consequently, CVD prevalence is anticipated to increase to 45% (>130 million) with higher projections for specific conditions like heart disease, heart failure and stroke [46]. Addressing issues that surround crash risk and decline, are crucial for aging in place and finding alternative transportation since older adults estimate they will live six years after driving cessation [47]. Conventional road tests and simulators are helpful with identifying specific deficits in performance on a controlled task. However, this generalizability to daily driving behavior is limited and only naturalistic data can provide insight into a driver’s native environment. The lack of studies employing naturalistic driving methodologies to measure everyday driving in CVD is partially surprising given their increasing use in studies of healthy older adults and those with preclinical and symptomatic AD [7,48,49,50]. A reason for this finding may be due to the ascent in technological development with in vivo vehicle tracking, data acquisition and transmission has only occurred in the past decade, while road tests and driving simulators have been a mainstay of driving outcome assessment for over 30 years. Another reason may be that road test and driving simulator outcomes offer investigators more control to examine driver performance metrics (e.g., lateral position on a road, gaze, reaction time) based on the study’s purpose. The handful of non-stroke studies substantiate the detrimental effect of heart failure, vascular dementia and WMH on driving performance and cessation. Future studies among older adults should endeavor to use naturalistic driving methodologies and interrogate the impact of heart failure, WMH/leukoaraiosis, vascular dementia, microbleeds and infarcts on daily driving. This would provide a distinct layer of ecologically grounded data on driving behavior instead of just performance and may help to pinpoint when changes in behavior occur over time signaling a need to intervene or facilitation driving retirement.

## 5. Conclusions

While no studies were found that examined naturalistic driving in older adults with non-stroke, vascular conditions, we were able to review how these conditions impact driving through other measurements. Heart failure, vascular dementia and white matter hyperintensities have all been studied to some extent in regard to older adult driving and all of these conditions increase the risk of poor driving and driving cessation. Additional studies are needed to look more deeply at driving behavior via naturalistic driving measurement, using in-vehicle data loggers and/or cameras. Furthermore, other conditions such as hypertension, hypercholesterolemia, myocardial infraction and atherosclerosis should be examined in this context. 

## Figures and Tables

**Table 1:** Publications included in systematic review.

First Author (Year)	Study Design	Purpose/Aim	Vascular Condition	Driving Outcome	N/Age Mean (SD)	Notable Findings
M. L. Alosco (2013)	Cross-sectional Case-control	Association between cognitive functioning and driving performance	Heart Failure	Simulator (STISIM: Build 2.08.03)	Cases N = 18:/67.7 (8.6) Controls N = 97:/19.9(3.0)	HF cases drove worse compared to controls; poor cognitive function was directly associated with poorer driving
M. L. Alosco (2015a)	Cross-sectional	Association between white matter hyperintensities and brain volume on driving	Heart Failure	Simulator (STISIM: Build 2.08.03)	N = 49:/69.1 (8.27)	MRI indices correlate with driving performance and cognitive functioning in patients with HF
M. L. Alosco (2015b)	Cross-sectional	Association between driving, physical fitness and cognitive functioning	Heart Failure	Simulator (STISIM: Build 2.08.03)	N = 18:/67.7 (8.6)	Poor physical fitness was associated with worse driving performance among patients with HF
B. A. Fausto (2017)	Retrospective Longitudinal	Whether heart failure predicts driving cessation	Heart Failure	Driving Habits Questionnaire Self-report	Cases N = 29:/74.7 (5.5) Controls N = 821/72.8 (5.4)	HF cases at a higher risk for driving cessation and may be mediated by poor cognitive functioning
L. J. Fitten (1995)	Cross-sectional	Examine driving performance between vascular and Alzheimer’s dementia	Vascular Dementia	Sepulveda Road Test	AD N = 13:/70.0 (7.4) VD N = 12:/71.8 (5.1)	The vascular dementia group showed greater variation in scores on the road test but higher cognitive scores compared to the AD group
M. Jang (2018)	Retrospective Longitudinal	Determine if WMH predicts driving cessation over time	WMH	Self-report Cessation	Mild N = 389:/66.0 (8.7) Mod. N = 116:/70.7 (7.4) Severe N = 35:/73.4 (6.4)	WMH and severity are associated with driving cessation and faster change in status from ‘currently driving’ to ‘no longer driving.’
K. Nakano (2014)	Cross-sectional	Association between WMH and driving performance	WMH	Standard licensing road test	Young: N = 9/24.7 (3.6) No WMH N = 13/70.0 (6.6) WMH N = 13/69.5 (6.1)	Both older groups performed worse that the young group. Older adults with WMH were distracted and made more errors on the road test
D Piersma (2018)	Cross-sectional	To examine prediction of fitness-to-drive among older adults with different dementia	Vascular Dementia	Road test and simulator (Jentig 50)	VD N = 12:/75.0 (5.3) FTD N = 14:/67.3 (10.3) DLB N = 8:/71.7 (10.3)	Compared to the other groups, the vascular dementia group made more errors on the road test and driving simulator and had poor cognitive functioning
R. V. Sims (2011)	Prospective Longitudinal	Whether heart failure predicts driving cessation	Heart Failure	Self-report Cessation	No HF N = 4544/73 (5) HF N = 839/76 (6)	HR is an independent risk factor for driving cessation over time while accounting for multiple covariates.
